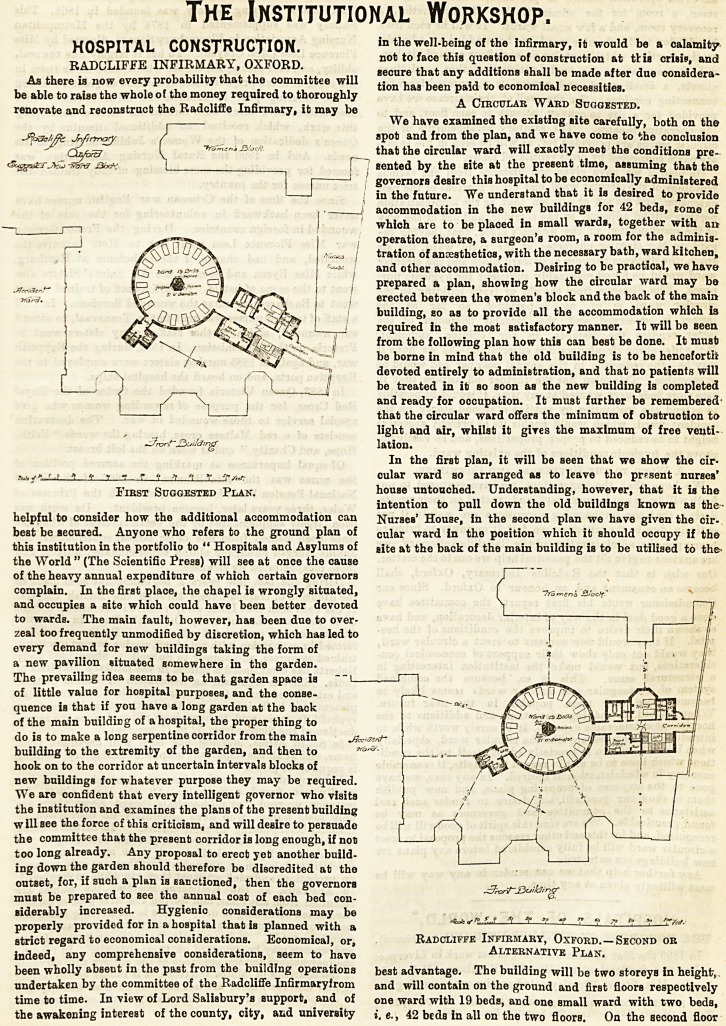# Radcliffe Infirmary, Oxford

**Published:** 1893-03-25

**Authors:** 


					Makch 25, 1893. THE HOSPITAL. 415
The Institutional Workshop.
HOSPITAL CONSTRUCTION.
RADCLIFFE INFIRMARY, OXFORD.
As there is now every probability that the committee will
be able to raise the whole of the money required to thoroughly
renovate and reconstruct the Radcliffe Infirmary, it may be
helpful to consider how the additional accommodation can
best be secured. Anyone who refers to the ground plan of
this institution in the portfolio to " Hospitals and Asylums of
the World " (The Scientific ?resa) will see at once the cause
of the heavy annual expenditure of which certain governors
complain. In the first place, the chapel is wrongly situated,
and occupies a site which could have been better devoted
to wards. The main fault, however, has been due to over-
zeal too frequently unmodified by discretion, which has led to
every demand for new buildings taking the form of
a new pavilion situated somewhere in the garden.
The prevailing idea seems to be that garden space is
of little value for hospital purposes, and the conse-
quence is that if you have a long garden at the back
of the main building of a hospital, the proper thing to
do is to make a long serpentine corridor from the main
building to the extremity of the garden, and then to
hook on to the corridor at uncertain intervals blockB of
new buildings for whatever purpose they may be required.
We are confident that every intelligent governor who visits
the institution and examines the plans of the present building
w ill see the force of this criticism, and will desire to persuade
the committee that the present corridor is long enough, if not
too long already. Any proposal to erect yet another build-
ing down the garden should therefore bo discredited at the
outBet, for, if such a plan is sanctioned, then the governors
must be prepared to see the annual coat of each bed con-
siderably increased. Hygienic considerations may be
properly provided for in a hospital that is planned with a
strict regard to economical considerations. Economical, or,
indeed, any comprehensive considerations, seem to have
been wholly abseut in the past from the building operations
undertaken by the committee of the Radcliffe Infirmaryfrom
time to time. In view of Lord Salisbury's Bupport, and of
the awakening interest of the county, city, and university
in the well-being of the infirmary, it would be a calamity
not to face this question of construction at ttis crisis, and
secure that any additions shall be made after due considera-
tion has been paid to economical necessities.
A Circular Ward Suggested.
We have examined the existing site carefully, both on the
spot and from the plan, and we have come to the conclusion
that the circular ward will exactly meet the conditions pre-
sented by the site at the present time, assuming that the
governors desire this hospital to be economically administered
in the future. We understand that it is desired to provide
accommodation in the new buildings for 42 beds, some of
which are to be placed in small wards, together with a?
operation theatre, a surgeon's room, a room for the adminis-
tration of anaesthetics, with the necessary bath, ward kitchen,
and other accommodation. Desiring to be practical, we have
prepared a plan, showing how the circular ward may be
erected between the women's block and the back of the main
building, so as to provide all the accommodation which is
required in the most satisfactory manner. It will be seen
from the following plan how this can best be done. It must?
be borne in mind that the old building is to be henceforth
devoted entirely to administration, and that no patients will
be treated in it so soon as the new building is completed
and ready for occupation. It must further be remembered'
that the circular ward offers the minimum of obstruction to
light and air, whilst it gives the maximum of free venti-
lation.
In the first plan, it will be seen that we show the cir-
cular ward so arranged as to leave the present nurses'
house untouched. Understanding, however, that it is the
intention to pull down the old buildings known as the-
Nurses' House, in the second plan we have given the cir-
cular ward in the position which it should occupy if the
site at the back of the main building is to be utilised to the>
beat advantage. The building will be two storeys in height,,
and will contain on the ground and first floors respectively
one ward with 19 beds, and one small ward with two beds,
i. e., 42 beds in all on the two floors. On the second floor
The Institutional Workshop.
HOSPITAL CONSTRUCTION. in the well-being of the infirmary, it would be a calamity
RADCLIFFE INFIRMARY, OXFORD. '? thia "l?"011 ?' ?onMrneti?n at tli. cri.i,, ud
. i. wKwrt.* ?i. **j. in secure that any additions shall bo made after due considera-
As there ,? bow every prob?bU,ty that the committee w II ^ hu ^ (<) oe(,<,sslUet.
be able to raise the whole of the money required to thoroughly
renovate and reconstruct the Radcliffe Infirmary, it may be A Circular ard Suggested.
.?  We have examined the existing site carefully, both on the
Jnfirmary ) spot and from the plan, and we have come to the conclusion
CtJbrt? I that the circular ward will exactly meet the conditions pre-
khSt-ttstg Bock 1   | ] sented by the site at the present time, assuming that the
governors desire this hospital to be economically administered
in the future. We understand that it is desired to provide
accommodation in the new buildings for 42 beds, some of
which are to be placed in small wards, together with an
operation theatre, a surgeon's room, a room for the adminis-
tration of anaesthetics, with the necessary bath, ward kitchen,
and other accommodation. Desiring to be practical, we have
prepared a plan, showing how the circular ward may be
erected between the women's block and the back of the main
building, so as to provide all the accommodation which is
required in the most satisfactory manner. It will be seen
from the following plan how this can best be done. It must
be borne in mind that the old building is to be henceforth
devoted entirely to administration, and that no patients will
be treated in it so soon as the new building is completed
and ready for occupation. It must further be remembered'
that the circular ward offers the minimum of obstruction to
light and air, whilst it gives the maximum of free venti-
JmrfJ3ai!<3ing lation.
In the first plan, it will be seen that we show the cir-
.r...? i <? t t r * * y cular ward so arranged as to leave the present nurses'
First Suggested Plan. house untouched. Understanding, however, that it is the
intention to pull down the old buildings known as the-
helpful to consider how the additional accommodation can Nurses' House, in the second plan we have given the cir-
best be secured. Anyone who refers to the ground plan of cular ward in the position which it should occupy if the
this institution in the portfolio to " Hospitals and Asylums of s|te at the back of the main building is to be utilised to the-
the World " (The Scientific Press) will see at once the cause
of the heavy annual expenditure of which certain governors
complain. In the first place, the chapel is wrongly situated,
and occupies a site which could have been better devoted
to wards. The main fault, however, has been due to over-
zeal too frequently unmodified by discretion, which has led to
every demand for new buildings taking the form of
a new pavilion situated somewhere in the garden.
The prevailing idea seems to be that garden space is
of little value for hospital purposes, and the conse-
quence is that if you have a long garden at the back
of the main building of a hospital, the proper thing to
do is to make a long serpentine corridor from the main
building to the extremity of the garden, and then to
hook on to the corridor at uncertain intervals blocks of
new buildings for whatever purpose they may be required.
We are confident that every intelligent governor who visits
the institution and examines the plans of the present building
w ill see the force of this criticism, and will desire to persuade
the committee that the present corridor is long enough, if not
too long already. Any proposal to erect yet another build-
ing down the garden should therefore bo discredited at the
outBet, for, if such a plan is sanctioned, then the governors -
must be prepared to see the annual coat of each bed con- m ^
siderably increased. Hygienic considerations may be
properly provided for in a hospital that is planned with a u??2?f????2?j???q_?5
strict regard to economical considerations. Economical, or, Radcliffe Infirmary, Oxford. ? Second or
indeed, any comprehensive considerations, seem to have Alternative Plan.
been wholly absent in the past from the building operations best advantage. The building will be two storeys in height,.
undertaken by the committee of the Radcliffe Infirmaryfrom and will contain on the ground and first floors respectively
time to time. In view of Lord Salisbury's support, and of one ward with 19 beds, and one small ward with two beds,
the awakening interest of the county, city, and university g,} 42 beds in all on the two floors. On the second floor
416 THE HOSPITAL, March 25, 1893.
an extra operation theatre is provided, with a surgeon's
room, a room for the administration of anaesthetics, a
recovery room, and a few small wards. It will be seen that,
in addition to the circular ward, we have added an adminis-
tration portion, which contains on each floor a ward kitchen,
a bath-room, stores for food, patients' clothes, and linen
closets, a small ward, and nurses' w.c., entered from the
connecting corridor. In the well of the staircase we have
provided for a lift to convey patients to each floor, and to
the operation theatre. The Banitary arrangements of the
circular wards are provided for, as will be seen in the separ-
ate parts connected by cross ventilated lobby.
On plan 2 we have given the distance from the various
buildings of the circular ward and other portions of the new
extension, and we are of opinion that, as the administtation
portion will be adjacent to the corridor, and out of the way
of the ward portion of the women's block, that, having re-
gard to the circular character of the main ward, the air
apace, the distance between each ward block and the circular
ward would be found reasonably sufficient for hygienic pur-
poses. The distance from the old or front building to the
circular ward is of no importance, so far as the
former is concerned, and the circular character of the
latter renders the distance, in our opinion, sufficient.
If the idea of the circular ward is entertained?as we hope it
may be?the reduction in the annual cost per bed would be
appreciable. This plan has the further advantage that in
case more beds were wanted at any time, it would be possible
to extend the institution by putting a second storey on the
accident ward, which would enable its at present excessive
height to be reduced to proper proportions, and so vastly im-
prove the hygienic conditions of the existing ward.
A Word to the Governors and Committee.
We hope that the suggestions we have ventured to put
forth will be taken in good part, and treated on their merits.
Seeing that the forthcoming extensions are mainly, if not
entirely, due to the criticisms we felt it our duty to bring to
bear upon the administration of the Radcliffa Infirmary, we
are anxious to give all the practical help we can in the matter.
Our wish is that the Radcliffe Infirmary, Oxford, shall
become an ornament and an honour to Oxford. Since our
Commissioner wrote his first report, the committee have
done a good deal in the way of interior decoration, and have
so shown their desire to improve the conditions of the hos-
pital. If they would now consent to erect a circular ward,
they would not only show their support of economical con-
siderations, but would make the institution interesting in
a structural sense. This is so, because the combined
system of rectangular and circular wards seems likely to
become more and more popular in the near future.
When Oxford is making alterations and additions to the
hospital at considerable expense, it is surely worth while to
introduce any new feature like the circular ward, especially
when it can be shown to precisely adapt itself to the condi-
tions which have to be met in regard to the site, if reasonable
economy in administration is desired. In any case, we have
gone to the expense of preparing plans, and now publish
them to show our goodwill, and desire to render such real
assistance to the committee and governors as may be
found practicable. We are sure this spirit of goodwill will be
recognised, and for this and other reasons the proposal to erect
a circular ward will be fully considered before any plans for
new buildings are accepted.
Any further help that we can render in any way will be
most willingly given at any time.

				

## Figures and Tables

**Figure f1:**